# Structure-based mutational analysis of ICAT residues mediating negative regulation of β-catenin co-transcriptional activity

**DOI:** 10.1371/journal.pone.0172603

**Published:** 2017-03-08

**Authors:** Mélanie J. Domingues, Juan Martinez-Sanz, Laura Papon, Lionel Larue, Liliane Mouawad, Jacky Bonaventure

**Affiliations:** 1 Institut Curie, PSL Research University, Bâtiment, Orsay, France; 2 University Paris-Sud, University Paris-Saclay, Orsay, France; 3 Inserm U1021, Normal and Pathological Development of Melanocytes, Orsay, France; 4 CNRS UMR 3347, Orsay, France; 5 Equipe Labellisée Ligue Contre le Cancer, Orsay, France; 6 Inserm, U1196, Chemistry, Modelling and Imaging for Biology, Orsay, France; 7 CNRS, UMR 9187, Orsay, France; University of Colorado Boulder, UNITED STATES

## Abstract

ICAT (Inhibitor of β-CAtenin and TCF) is a small acidic protein that negatively regulates β-catenin co-transcriptional activity by competing with TCF/LEF factors in their binding to β-catenin superhelical core. In melanoma cells, ICAT competes with LEF1 to negatively regulate the *M-MITF* and *NEDD9* target genes. The structure of ICAT consists of two domains: the 3-helix bundle N-terminal domain binds to β-catenin Armadillo (Arm) repeats 10–12 and the C-terminal tail binds to Arm repeats 5–9. To elucidate the structural mechanisms governing ICAT/β-catenin interactions in melanoma cells, three ICAT residues Y15, K19 and V22 in the N-terminal domain, contacting hydrophobic β-catenin residue F660, were mutated and interaction was assessed by immunoprecipitation. Despite the moderate hydrophobicity of the contact, its removal completely abolished the interaction. In the ICAT C-terminal tail consensus sequence, neutralization of the electrostatic interactions between residues D66, E75 and β-catenin residues K435, K312, coupled to deletion of the hydrophobic contact between F71 and β-catenin R386, markedly reduced, but failed to abolish the ICAT-mediated negative regulation of *M-MITF* and *NEDD9* promoters. We conclude that in melanoma cells, anchoring of ICAT N-terminal domain to β-catenin through the hook made by residue F660, trapped in the pincers formed by ICAT residues Y15 and V22, is crucial for stabilizing the ICAT/β-catenin complex. This is a prerequisite for binding of the consensus peptide to Arm repeats 5–9 and competition with LEF1. Differences between ICAT and LEF1 in their affinity for β-catenin may rely on the absence in ICAT of hydrophilic residues between D66 and F71.

## Introduction

The canonical Wnt/β-catenin signaling pathway is involved in multiple normal and pathological biological processes. This pathway is frequently altered in diverse cancers including carcinoma, pseudopapillary tumors and melanoma [1–4]. In melanoma, two of the main targets of the Wnt/β-catenin pathway are the promoters of *M-MITF* and *NEDD9* genes. The melanocyte specific isoform of the transcriptional factor M-MITF (MIcrophtalmia-associated Transcription Factor) [1, 5] is essential for melanogenesis, and involved in melanoma formation and progression [6, 7]. Variable M-MITF expression has been reported in melanoma cell lines, often correlating with their malignancy [8, 9]. NEDD9 (Neural precursor cell Expressed Developmentally Down-regulated 9) is an adaptor protein transducing signals and playing a key role in cell proliferation and migration. NEDD9 overexpression in human metastatic melanoma is frequent [10] and the transcriptional activation of the gene was recently found to be β-catenin-dependent [11, 12].

In the presence of a Wnt signal, the multifunctional protein, β-catenin, is translocated to the cell nucleus where it interacts with TCF/LEF transcription factors, displacing the Groucho repressor [13] and activating multiple target genes [2, 14, 15]. Both positive and negative regulators of the Wnt/β-catenin signaling pathways have been identified including Cadherins, TCFs, Adenomatous Polyposis Coli (APC), Axin and the transcriptional inhibitor ICAT. This highly conserved small protein of 81 amino-acids is encoded by the vertebrate-specific *CTNNBIP1* gene [16]. ICAT transcripts are ubiquitously expressed during embryonic development [17]. At the cellular level, ICAT localizes mostly in the cytoplasm but also in the nuclei of normal and cancer cells, sign of a dynamic distribution between the two compartments [12,18]. In the nuclear compartment of colorectal cancer cells, ICAT competes with TCF4 (now referred to as TCF7L2) for binding to β-catenin, thus negatively regulating its co-transcriptional activity [16]. In a panel of human melanoma cell lines we have recently demonstrated variable ICAT expression. High levels of ICAT transcripts were found in metastatic cells with a strong motility, while non-metastatic cells expressed low ICAT mRNA amounts and migrated poorly [12]. Differences in cell migration were ascribed to ICAT-dependent negative regulation of NEDD9 expression. Investigating how ICAT is able to modulate *M-MITF* and NEDD9 expression in melanoma cells should help clarifying the mechanisms of interaction between ICAT and β-catenin.

β-catenin functions as a scaffold for multiprotein assemblies [15]. It is a 781 amino-acid protein with a central core region composed of 12 Armadillo (Arm) repeats, each one (except repeat 7) consisting of three α helices. Arm repeats 5–9 form a positively charged groove involved in the binding of several ligands [19]. The crystal structure of the full-length zebrafish β-catenin [20] has allowed the identification of an additional α helix C which docks onto the third helix of Arm repeat 12 through three highly conserved leucine residues (S1 Fig). This helix C has been suggested to mediate the interaction with ICAT [20], however, its precise role is still unknown since its deletion in cultured cells did not affect β-catenin turnover [21].

**Fig 1:**
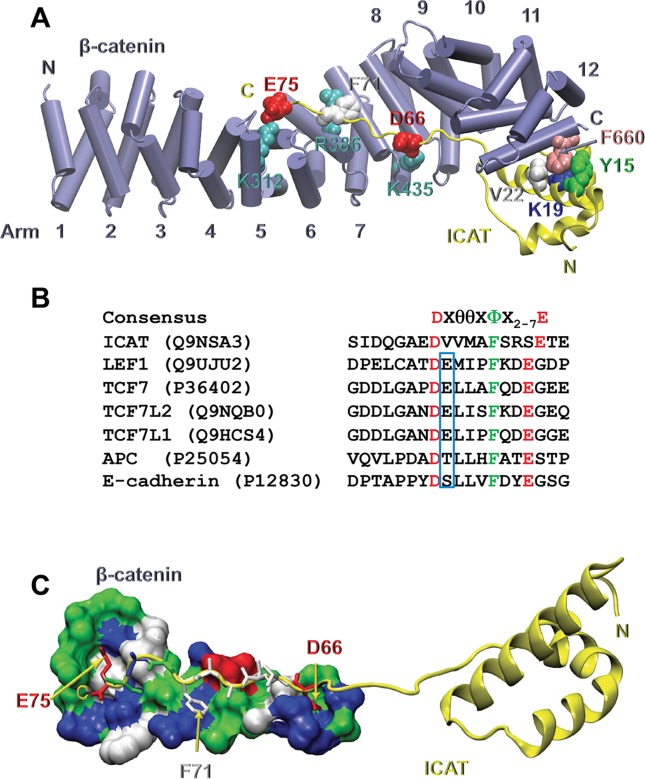
Crystal structure of the β-catenin/ICAT complex. **A** Crystal structure of ICAT bound to the core domain of β-catenin (PDB code 1LUJ,[22]). ICAT is shown as yellow ribbons and β-catenin as purple cylinders. The secondary structures were calculated using the program STRIDE [37]. Residues mutated in this study are shown as hard spheres. ICAT residues are colored according to their characteristics: white for hydrophobic, green for polar, red for acidic and blue for basic residues. β-catenin F660 is in pink and the basic residues facing the C-terminal domain of ICAT are in cyan. **B**. Sequence alignment of the consensus peptide from several β-catenin binding proteins. The conserved acidic residues are in red and the aromatic residue in green. The first X residues, when they are hydrophilic, are boxed. **C**. β-catenin/ICAT complex showing the interaction between ICAT consensus peptide of the C-terminal domain (ribbon and sticks) and its facing β-catenin residues (surface). All residues are colored according to their characteristics. Figures were drawn using VMD software [38].

Crystal structures of the ICAT/β-catenin complex at 2.5Å (PDB: 1LUJ, [22]) and 2.1Å resolution (PDB: 1M1E, [23]) have shown that the two proteins interact in an anti-parallel orientation (Fig 1A). Two main regions in ICAT have been identified: i) the N-terminal domain consists of a bundle of three α helices, with helix 1 (H1) directly interacting with Arm repeats 10–12 of β-catenin; ii) the C-terminal domain is intrinsically unstructured but adopts a β-strand like structure when bound to β-catenin Arm repeats 5–9. The latter domain contains a β-catenin peptide-binding motif DXθθXΦX_2-7_E, where θ is an aliphatic hydrophobic amino acid, Φ an aromatic residue (mainly Phenylalanine) and X_2-7,_ two to seven variable residues (Fig 1B). This consensus motif is present in several β-catenin ligands including E-cadherin, APC, Axin and members of the TCF/LEF family, to mediate their interaction with the groove of β-catenin [24–26]. Therefore, the competition between ICAT and these proteins likely occurs in this region. Slight variations in the consensus sequence may account for differences in ligand binding affinities [27], although the exact contribution of residues D, Φ and E in conferring binding energy to the groove remains debated. Indeed, in TCF7L2, residue F21 was found to be dispensable in one study [28] or essential in another one [29] for its binding to β-catenin. Additionally, the disruption of the salt bridge between residue E24 in TCF7L2 and K312 in β-catenin, only mildly affected the affinity [28]. Therefore, the contribution of these residues to the interaction of ICAT with β-catenin needs clarification.

Based on the crystal structure of the β-catenin/ICAT complex [22, 23] several contacts between the ICAT N-terminal domain and β-catenin were assumed to stabilize the two proteins interaction. This included the hydrophobic contacts between ICAT residues Y15, K19 and V22 and β-catenin F660. However, evaluation of their relative contribution is required.

To investigate the importance of the highlighted residues for the ICAT/β-catenin interaction in melanoma cells, point mutations were introduced in ICAT and β-catenin by site-directed mutagenesis. We selected six highly conserved ICAT residues, three being located in the N-terminal domain, and three in the C-terminal domain consensus peptide (Fig 1 and S2 Fig). We also mutated their facing residues in β-catenin. The consequences of these mutations on the ICAT/β-catenin complex and the transcriptional regulation of *M-MITF* and *NEDD9* promoters were studied in two melanoma cell lines expressing opposite amounts of ICAT and β-catenin.

## Materials and methods

### Cell culture

The human melanoma cell lines Lu1205 and Mel501, obtained respectively from Dr M. Herlyn (Wistar Institute, Philadelphia, PA, USA) and C. Goding (Ludwig Institute, Oxford UK) were maintained in RPMI 1640 medium (Gibco) supplemented with 10% foetal bovine serum (Sigma), 5 mM L-glutamine, 100 U/mL penicillin and 100μg/mL streptomycin. All cells were grown at 37°C in a humidified atmosphere containing 5% carbon dioxide.

### Plasmids, cDNA constructs and mutagenesis

The human ICAT cDNA (243bp) cloned in the pFLAG-CMV2 expression vector was kindly provided by Dr. C. Gottardi (University of Chicago IL, USA). The expression of ICAT being under the control of the CMV promoter, the vector is referred to as CMV::ICAT WT in the manuscript. A triple mutant form of ICAT in helix 1 (ICAT-DQE mutant) derived from the pFLAG-ICAT-CMV2 construct was created with primers shown in S1 Table. The forward primer LL1806 containing three nucleotide changes (shown in red) when compared to the wild-type sequence was coupled with the reverse primer LL1521. A 186 bp PCR fragment was generated and restriction digested with BspEI and BlpI. This 128 bp sub-fragment was exchanged with the equivalent wild-type fragment isolated from the pFLAG-ICAT-CMV2 vector. Single (D66G, F71A, E75V), double (D66G + E75V = GV; F71A+ E75V = AV and D66G + F71A = GA) and triple (D66G + F71A + E75V = GAV) mutants of ICAT C-terminal domain were created using the Quick-Change Site Directed Mutagenesis kit (Stratagene) and primers listed in S1 Table. The HA-tagged CMV::β-catenin-NLS expression vector was used for overexpression of β-catenin in the nuclei of transfected cells [1]. Single mutants of the HA tagged CMV::β-catenin-NLS vector (K312E, K435E, R386G, F660S, F660A) and the truncated mutant Δ665 (premature stop codon at residue 665) were also created by site directed mutagenesis using primers listed in S1 Table. All constructs were sequenced to validate presence of the different mutations. The *M-MITF*::*Luc* promoter (-2293 to + 120) in pGL3 and the *NEDD9*::*Luc* promoter (-2011 to + 72) in pGL3 were kindly provided by Dr C. Goding (Ludwig Institute, Oxford UK) and Dr W. Liu (Louisiana State University Health Sciences Center, New Orleans, LA, USA), respectively.

### Production of recombinant wild-type (WT) and mutant ICAT-GST proteins

The WT human ICAT cDNA cloned in the bacterial expression vector pGEX-4T3 was provided by C. Gottardi (USA). Mutant cDNAs including ICAT-DQE, ICAT-GV, ICAT-AV, ICAT-GA and ICAT- GAV were subcloned in the pGEX-4T3 vector and used to transform *E*.*coli* BL21 competent cells (Stratagene). Positive clones were grown at 37°C for 2–3 hrs to an OD_600_ of 0.6–0.8 and protein synthesis was induced with 0.5 mM IPTG at 37°C for 3–4 hrs. The bacterial cultures were centrifuged and the cell pellets sonicated. Bacterial lysates were incubated with glutathione Sepharose 4B beads (GE Healthcare) overnight at 4°C. The beads were washed twice with 1X PBS, 0.3M NaCl, Triton 0.1%, protease inhibitors, twice with the same buffer containing 0.15M NaCl, and once with 1X PBS, Triton 0.1%. After re-suspension in one volume of 1X PBS, aliquots were analyzed on polyacrylamide gels and stained with Coomassie blue. A similar procedure was used to produce recombinant human LEF1-GST protein.

### Purification and Circular Dichroism (CD) analysis of ICAT WT and DQE recombinant proteins

ICAT WT and ICAT-DQE recombinant proteins were purified from their GST forms. Cleavage of the GST tag was achieved by the addition of thrombin (1U/μl in PBS) and incubation at room temperature for 16h. Solutions containing the cleaved proteins were loaded on Hi-Trap Benzamidine Sepharose 4 Fast Flow columns (GE-Healthcare) and eluted with phosphate buffer 20mM, 0.15M NaCl, pH 7.3. Collected fractions were spin concentrated on Amicon Ultra 3K centrifugal filter (Millipore) and dialyzed against 10mM Phosphate buffer pH 7.0 containing 100 mM ammonium sulfate. Final concentrations were determined by UV absorption at 280 nm. Aliquots were separated on polyacrylamide gels and stained with Coomassie blue.

CD spectra were recorded using a J-710 Jasco spectro-polarimeter. Cuvettes with a 1 mm path length were used. Each spectrum was averaged using eight accumulations, a 2nm bandwidth and a scan speed of 50 nm/min at 20°C. The melting curves of ICAT-WT and ICAT-DQE were established by monitoring the ellipticity [θ]_λ_ at a fixed wavelength of 222 nm. Temperature ranged from 20°C to 90°C with a slope of 1°C/min. The CDSSTR algorithm was used to deconvolute the CD spectra and estimate the percentage of α-helix content [30]; http://lamar.colostate.edu/~sreeram/CDPro/ListPro.htm)

### Western blotting, immunoprecipitation and affinity precipitation

Total cell lysates (TCL) were prepared as described earlier [12] and submitted to WB analysis. Immunoprecipitation (IP) was achieved by incubating TCL with an anti-Flag monoclonal antibody for 2hrs at 4°C. Immune complexes were bound to Protein G agarose beads. After washing with RIPA, beads were boiled for 10 min in loading buffer. For affinity precipitation, TCL in RIPA (500 μg of total proteins) were incubated for 3 hrs at 4°C with 20–30 μL of a 50% suspension of WT or mutant ICAT-GST, or LEF1-GST recombinant proteins coupled to glutathione-Sepharose 4B beads. Affinity precipitated proteins were washed five times and subjected to SDS-PAGE and immuno-blotting analysis. Gel densitometry was performed using ImageJ. Control values were arbitrarily fixed to 1.0.

### Antibodies and cell immunofluorescence

The previously described rabbit polyclonal antibody raised against a synthetic ICAT peptide was used for WB [12]. Mouse monoclonal antibodies against human ICAT (clone 5C6), anti-Flag M2 and anti-β-actin (clone AC-15) were obtained from Sigma-Aldrich. Mouse anti-HA monoclonal antibody (clone 12CA5) was from Roche Life Science. Rabbit polyclonal antibody raised against β-catenin was from Abcam. The rabbit polyclonal anti-p27^kip1^ antibody and the rabbit monoclonal antibody (clone D13A1) raised against the non-phospho (active) β-catenin S33/37,T41, were obtained from Cell Signaling. The mouse anti-LEF1 monoclonal antibody (clone 2D12) was from Millipore. The rabbit polyclonal anti-MITF antibody was a gift from Dr S. Saule (Institut Curie, Orsay). Immunofluorescent staining of cultured cells fixed in 4% PFA was achieved as described [12].

### Transfections and dual luciferase assays

Lipofectamine 2000 (Life Technologies) was used to transiently co-transfect cells with the *M-MITF* or *NEDD9* promoters and expression vectors for LEF1, β-catenin (WT and mutants) and/or ICAT (WT and mutants). The pRL-TK *Renilla* luciferase reporter construct was used as an internal control for transfection efficiency. Forty-eight hrs post-transfection, cells were lysed with passive lysis buffer and luciferase activities (*Firefly* and *Renilla*) were measured using the Dual Luciferase reporter Assay (Promega, Madison WI) in a TriStar luminometer (Berthold, Germany). *Firefly* luciferase activity was normalized to *Renilla* luciferase activity.

### Statistical analysis

Prism6 software (GraphPad, La Jolla, CA) was used for statistical analyses. Luciferase activity differences were assessed using two-way analysis of variance (ANOVA) test followed by Tukey’s post-test.

## Results

### β-catenin is the main ICAT interactor in melanocytic cells

So far, β-catenin is the only demonstrated protein known to interact directly with ICAT at the physiological level [18, 12]. However, ICAT may also bind with high or mild affinity to other proteins. To identify interactors in the melanocyte lineage, a yeast two-hybrid screen using human ICAT as bait was performed with a human melanocyte cDNA library as prey. Although various putative partners were detected at low frequencies by sequencing of prey clones, most of the positive clones (80%) corresponded to CTNNB1/β-catenin cDNAs (S2 Table). These results were compared to the interaction data originating from a large-scale mapping in HEK cells, using a mass spectrometry-based approach [31]; S3 Table). Only two similar proteins, β-catenin and γ-catenin/plakoglobin, were identified in both studies. That ICAT interacts with both Plakoglobin and β-catenin is not unexpected owing to the strong conservation of their Armadillo domains [32]. However β-catenin got the highest confidence score, suggesting a higher affinity for ICAT than plakoglobin. We concluded that β-catenin is the main ICAT-interacting protein in cells of the melanocyte lineage.

### Mel501 and Lu1205, melanoma cell lines with opposite ICAT and β-catenin protein levels

Two melanoma cell lines, the non-metastatic Mel501 and the highly metastatic Lu1205, expressing 3 times more ICAT than Mel501 [12] were chosen to assess the effect of ICAT on the co-transcriptional activity of β-catenin. Using WB, we found that in Mel501 cells, total β-catenin was 4 fold more abundant than in Lu1205 cells. Immunofluorescence (IF) staining with an antibody (here referred to as ABC), directed against the active signaling form of β-catenin showed a faint nuclear signal in Lu1205 cells and a strong signal in Mel501 cells. The amount of ABC in Mel501 cells was 7 fold higher than in Lu1205 cells (S3A Fig, S4 Table), indicating constitutive activation of the canonical Wnt/β-catenin pathway in Mel501. Based on these data and in order to minimize the role of the endogenous proteins, Mel501 cells exhibiting a low level of endogenous ICAT were used to assess the consequences of ectopically expressed ICAT mutants. Lu1205 cells exhibiting a low amount of nuclear β-catenin were chosen for the analysis of overexpressed NLS-tagged β-catenin mutants.

### In melanoma cells, ICAT negatively regulates M-MITF expression in a dose-dependent manner

**Fig 2:**
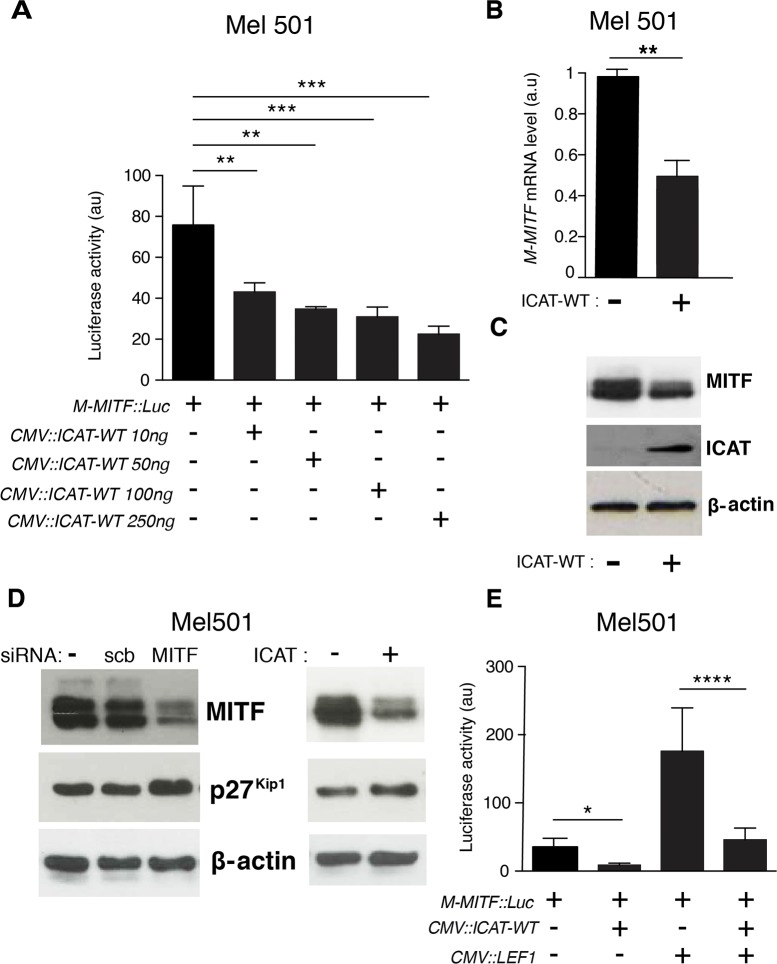
ICAT negatively regulates the M-MITF promoter activity by competing with LEF1. **A.** Mel501 cells were transfected with a *M-MITF*::*luciferase* vector in the presence of increasing amounts of *CMV*::*ICAT-WT* expression vector. Data are presented as means ± SEM of three independent experiments. **B.** qRT-PCR analysis of *M-MITF* mRNA levels in Mel501 cells transfected with empty or *ICAT-WT* expression vectors. **C.** WB analysis of MITF and ICAT proteins in Mel501 cells transfected with empty or *ICAT-WT* expression vectors. **β**-actin = loading control. **D.** WB analysis of MITF and p27^Kip1^ protein levels in siRNA and ICAT-transfected Mel501 cells. SiMITF treatment and ICAT overexpression induce respectively a 42% and 35% increase of p27 protein amount; Scrb = control scrambled siRNA. **E**. Mel501 cells were transfected with *M-MITF*::*luciferase*, *LEF1* and *ICAT-WT* expression vectors. Data are presented as means ± SEM of three independent experiments. *p<0.05, **p<0.01, ***p< 0.001; ****p<0.0001.

*M-MITF* being a master regulator gene of the melanocyte lineage and melanomagenesis, but also a target of the TCF/LEF/β-catenin complex, we evaluated by WB and IF the expression of MITF in our cell lines. High protein amount was found in the nuclei of Mel501 cells, while in Lu1205 cells, MITF was undetectable (S3B Fig, S4 Table). The *M-MITF* promoter comprises three TCF/LEF binding sites. To test whether the low level of MITF in Lu1205 cells was related to the inhibitory effect of endogenous ICAT, its level in Mel501 cells was ectopically increased and M-MITF expression was evaluated by four different approaches. 1) Mel501 cells were co-transfected with the *M-MITF*::*luciferase* reporter construct and increasing amounts of *CMV*::*ICAT* expression vector. The luciferase (luc) activity showed a dose-dependent negative effect of ICAT (Fig 2A). Based on this result, a dose of 250 ng of *CMV*::*ICAT* plasmid was used in subsequent experiments. 2) Quantitative RT-PCR in Mel501 cells transiently transfected with *ICAT-WT* showed a 50% reduction of *M-MITF* mRNA amount (Fig 2B), revealing the inhibitory transcriptional effect of exogenous ICAT on *M-MITF* expression. 3) WB analysis showed a marked reduction of MITF protein in the presence of ICAT (Fig 2C). 4) The effect of variable MITF levels on the amount of cyclin-dependent kinase inhibitor, p27^Kip1^, was used to verify the action of ICAT. Transient ectopic expression of ICAT-WT in Mel501 coordinately reduced MITF and increased p27^Kip1^ protein levels (Fig 2D). This result is consistent with the previous finding that siMITF treatment increased p27^Kip1^ expression [33]. Collectively, these data demonstrate a negative regulatory effect of ICAT on *M-MITF* expression in Mel501 cells. The lack of detectable MITF protein in Lu1205 cells could be ascribed, at least in part, to their high level of endogenous ICAT.

### ICAT competes with LEF1 to bind nuclear β-catenin in melanoma cells

In colon cancer cells, ICAT was found to compete with TCF7L2 for binding to β-catenin [16]. In Mel501 and Lu1205 cells, qRT-PCR evaluation of *TCF7L2* transcripts disclosed an almost undetectable mRNA level, whereas *LEF1* mRNA was much more abundant especially in Lu1205 where its amount was twice higher than in Mel501 (S3C Fig). This result was confirmed at the protein level (S3D Fig). Since the biological functions of LEF1 and TCF7L2 are not fully redundant [34], competition between ICAT and LEF1 for regulating transcriptional activity was tested by co-transfecting Mel501 cells with *CMV*::*ICAT*, *CMV*::*LEF1* and *M-MITF*::*luc* vectors. LEF1 overexpression greatly increased luciferase activity of the *M-MITF*::*luc* promoter. This stimulating effect was almost completely abrogated in the presence of exogenous WT ICAT, illustrating the competition between the two β-catenin ligands (Fig 2F).

### Mutations in the ICAT N-terminal domain prevent the interaction with β-catenin

In an attempt to suppress the binding of ICAT to β-catenin without impairing the intrinsic secondary structure of ICAT, three residues (Y15, K19 and V22) in the helix1 of ICAT N-terminal domain were mutated simultaneously. Aromatic residue Tyrosine 15 was converted into Aspartate (Y15D). The positively charged Lysine 19, which contributes to the hydrophobic packing through the long aliphatic part of its side chain, was mutated into Glutamine, a shorter neutral residue (K19Q). Valine 22 was converted into Glutamate, a negatively charged residue (V22E). The resulting mutant is referred to as ICAT-DQE (Table 1). To determine whether the triple mutation critically affected the secondary structure of the protein, recombinant ICAT-WT and ICAT-DQE were purified from their GST fusion forms (S4A Fig), and their Circular Dichroism (CD) spectra were recorded. The two spectra were similar (S4B Fig) and their deconvolution with the CDSSTR algorithm showed that both proteins were composed of about 40% of α-helices and 17% of β-sheet, in good agreement with the ICAT crystal structure. This indicates that the three mutations did not significantly affect the secondary structure of the isolated protein. Stability of the two proteins was further evaluated by thermal denaturation using CD at 222 nm. Melting temperature (Tm) derived from the curves was 55°C for both the WT and mutant proteins (S4C Fig), showing that these mutations did not alter the thermal stability.

**Table 1:** List of ICAT and β-catenin mutants created by site directed-mutagenesis.

Gene	Protein	Mutation
CTNNBIP1	ICAT	Y15D + K19Q + V22E (DQE)*
CTNNBIP1	ICAT	D66G
CTNNBIP1	ICAT	F71A
CTNNBIP1	ICAT	E75V
CTNNBIP1	ICAT	D66G + F71A (GA)*
CTNNBIP1	ICAT	D66G + E75V (GV)*
CTNNBIP1	ICAT	F71A + E75V (AV)*
CTNNBIP1	ICAT	D66G + F71A + E75V (GAV)*
CTNNB1	β-catenin	F660S ; F660A
CTNNB1	β-catenin	K312E
CTNNB1	β-catenin	K435E
CTNNB1	β-catenin	R386G
CTNNB1	β-catenin	D665X (Δ665)*

* Abbreviations used in the text referring to mutants.

**Fig 3:**
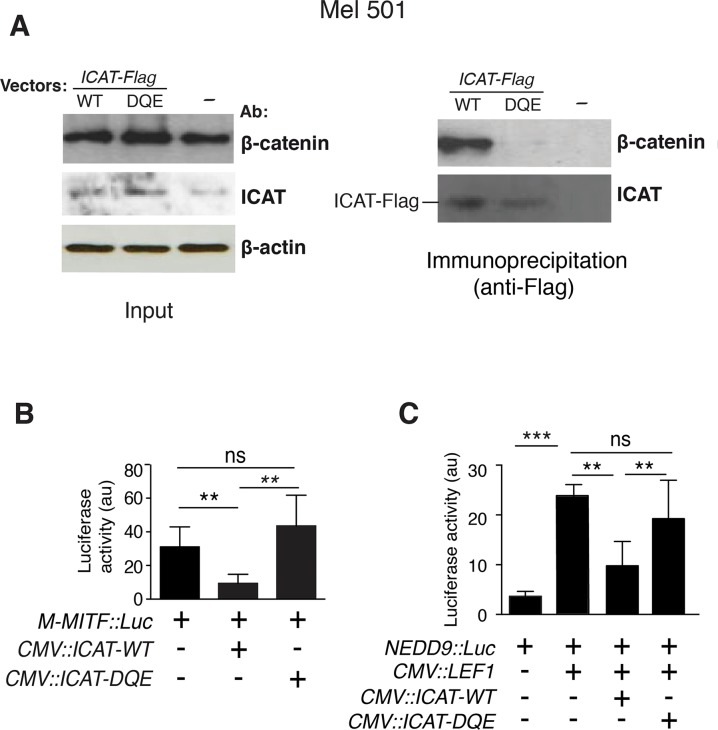
Mutations in the ICAT N-terminal domain prevent binding to β-catenin and abolish the negative regulation of the *M-MITF* and *NEDD9* promoter activities. **A**. Left: WB analysis of Mel501 cell lysates (Input), β-actin = loading control; right: immunoprecipitation with an anti-Flag antibody of endogenous β-catenin in Mel501 cells overexpressing ICAT-WT or ICAT-DQE. **B**. Cells were transiently transfected with *M-MITF*::*luciferase*, *ICAT-WT* or *-DQE* expression vectors. (C) Cells were transfected with *NEDD9*::*luciferase*, *ICAT-WT* or *-DQE* and *LEF1* expression vectors. **p<0.01, ***p< 0.001, ns = non significant

To test whether residues Y15, K19 and V22, were required for ICAT binding to β-catenin, we transfected Mel501 cells with WT or mutant (DQE) *ICAT-Flag* vectors and performed IP assays (Fig 3A). As expected, exogenous ICAT-WT protein bound efficiently to endogenous β-catenin present in Mel501 cell lysates, while ICAT-DQE mutant failed to immunoprecipitate β-catenin, revealing a loss of function of this mutant. In a reporter luciferase assay using the *M-MITF* promoter, ectopic expression of ICAT-DQE had no effect on *M-MITF* luc activity in Mel501 cells, both in the presence and absence of exogenous LEF1 (Fig 3B and 3C).

### Interaction between β-catenin and ICAT relies on β-catenin residue F660

**Fig 4:**
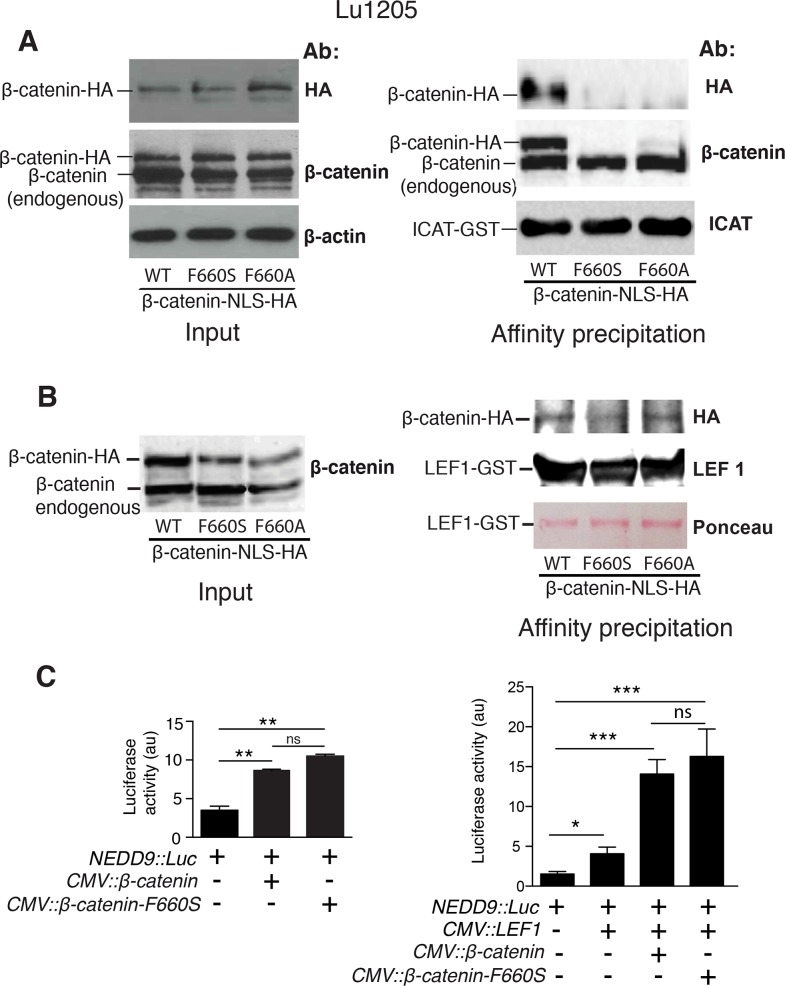
β-catenin residue F660 is critical for ICAT anchoring to Arm repeat 12 but plays no role in the affinity for LEF1. **A**. Left: Western blot (WB) analysis of lysates (Input) from Lu1205 cells transfected with WT or mutant HA-tagged β-catenin constructs; right: pull-down assay of β-catenin WT and mutants F660S and F660A by WT ICAT-GST recombinant protein. **B**. Left: Input from Lu1205 cells transfected with WT or mutant HA-tagged β-catenin constructs; right: pull-down assay of β-catenin WT and mutants F660S and F660A by LEF1-GST recombinant protein. **C**. Lu1205 cells were transiently transfected with a *NEDD9*::*luciferase* vector. These cells were also transfected with either *NLS-*β*-catenin-WT* or *NLS-*β*-catenin-F660S* expression vectors in the absence (left) or presence (right) of exogenous LEF1. Data are presented as means ± SEM of three independent experiments. *p<0.05, **p<0.01, ***p<0.001, ns = not significant.

The requirement of the three ICAT residues mutated above for proper interaction with β-catenin may be ascribed to their contact with the facing residue F660 (S2 Fig), as indicated by the crystal structure of the complex [22]. However, four other β-catenin residues are in close contact with one or the other of the three ICAT residues, and the electrostatic interaction between β-catenin E664 and ICAT K19, may contribute to the affinity. Consequently, β-catenin residue F660 was mutated into serine (F660S) or alanine (F660A) (Table 1) to evaluate its contribution to the interaction with ICAT in Lu1205 cells. The β-catenin F660S mutant failed to interact with ICAT, whereas the interaction with the F660A mutant was impaired but not totally abolished (Fig 4A). By contrast, both β-catenin mutants interacted normally with the recombinant LEF-GST protein (Fig 4B). Since WT β-catenin is a co-transcriptional activator of *NEDD9* [12], we tested using a reporter assay whether the activity of the *NEDD9* promoter in Lu1205 cells was affected by the F660S mutation. The activating role of β-catenin F660S in the absence or presence of ectopic LEF1 was similar to WT (Fig 4C). Thus, residue F660 seems dispensable for the interaction with LEF1, although crucial for the interaction with ICAT.

### β-catenin helix C is dispensable for ICAT/β-catenin interaction

**Fig 5:**
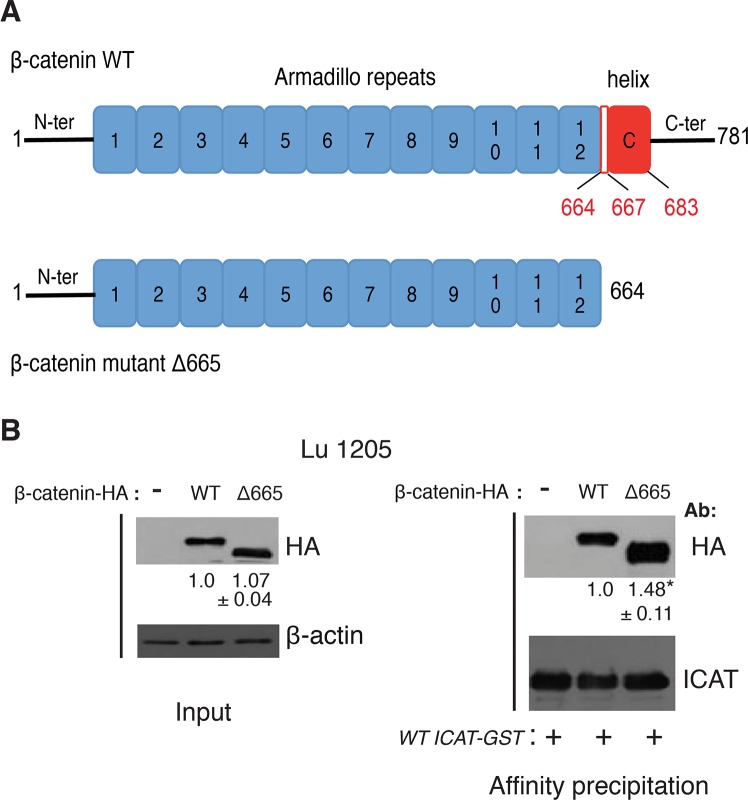
The lack of helix C in β-catenin does not prevent interaction with ICAT. **A**. Schematic representation of WT and mutant Δ665 HA-tagged β-catenin-NLS proteins. **B**. Total cell lysates from Lu1205 cells transfected with WT or mutant β-catenin Δ665 were analyzed by WB or affinity immunoprecipitated with WT ICAT-GST recombinant protein and blotted with anti-HA and anti-ICAT antibodies. Numbers represent mean ± SD of normalized densitometry values from three independent experiments, *p<0.05.

Implication of the β-catenin helix C, spanning conserved residues 667–683 in the C-terminal domain (S1 Fig), in increasing the interaction with ICAT N-terminal domain has been hypothesized [20], despite its absence in the crystal structures of the β-catenin/ICAT complex [22, 23]. To test this assertion, we generated a β-catenin truncated protein by introducing a premature stop codon at position 665 (Fig 5A). The Δ665 HA-tagged β-catenin mutant was properly expressed in Lu1205 cells at the same level as the WT. It interacted with the recombinant WT ICAT-GST protein more efficiently (x1.5) than the full-length WT β-catenin (Fig 5B). Contrary to the model prediction [20], helix C appeared dispensable for an efficient interaction of ICAT with β-catenin Arm repeats 11–12. The observed increased affinity is in keeping with results from Mo et al [21], suggesting that the absence of the unstructured β-catenin C-terminal domain that normally extends toward the N-terminal Arm repeats, could facilitate the recruitment of various ligands. Moreover, the close contact of helix C with residue F660 (S1 Fig) could decrease the affinity of ICAT helical bundle for Arm repeats 11–12.

### Mutations in the ICAT consensus peptide reduce but do not abolish its affinity for β-catenin

**Fig 6:**
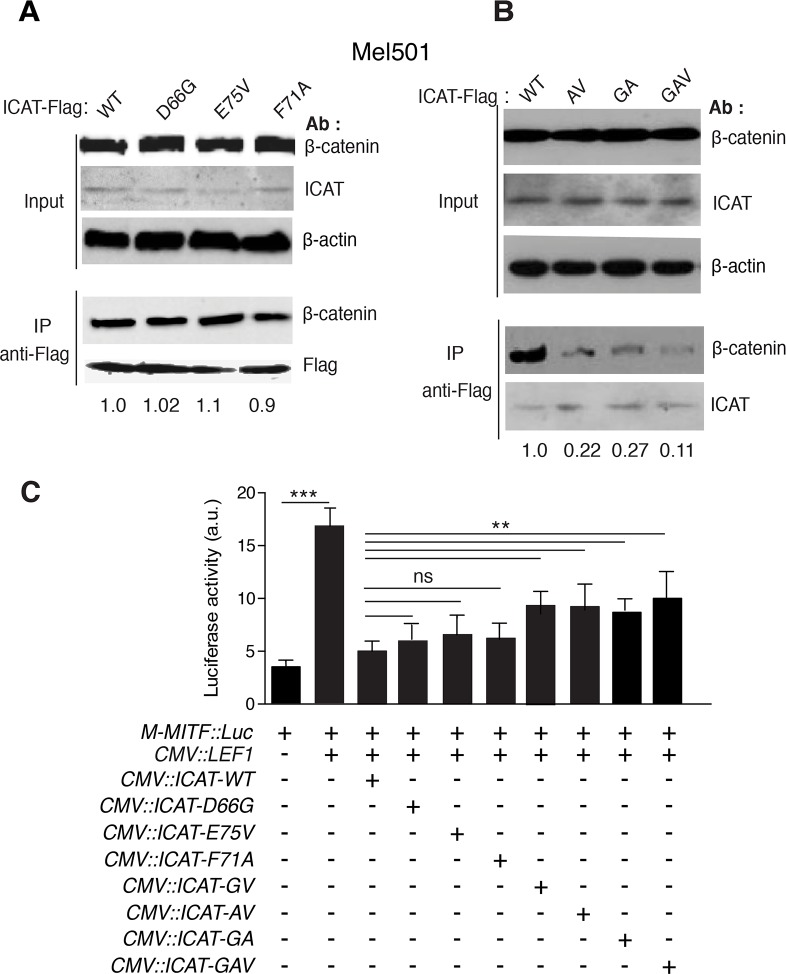
Effect of mutations in the C-terminal domain on ICAT binding to β-catenin and regulation of *M*-*MITF* promoter activity. **A**. Upper: WB analysis of cell lysates (Input) from Mel501 cells transiently transfected with WT ICAT or ICAT single mutants (D66G, E75V and F71A); Lower: Immunoprecipitation (IP) of endogenous β-catenin by WT and single ICAT-Flag mutants. **B** Upper: WB analysis of cell lysates (Input) from Mel501 cells transiently transfected with WT ICAT or ICAT double (AV, GA) or triple (GAV) mutants. Lower: IP of endogenous β-catenin by WT and double or triple ICAT-Flag mutants. Numbers under each lane represent normalized densitometry values. β-actin = loading control. **C**. Mel501 cells were transiently co-transfected with *M-MITF*::*luciferase*, *LEF1*, Flag-tagged *ICAT WT* and *ICAT* mutants (*D66G; E75V; F71A; D66G*,*E75V; F71****A***,*E75****V****; D66****G***,*F71****A****; D66****G***,*F71****A***,*E75****V***) expression vectors. Data are presented as means ± SEM of three independent experiments. *p<0.05, **p<0.01, ***p<0.001, ns = not significant.

Based on sequence alignments, the three conserved residues D66, F71 and E75 in the ICAT consensus peptide (Fig 1B), seemed important for the interaction with β-catenin Arm repeats 5–9 (Fig 1C). According to the crystal structure, residues D66 and E75 formed salt bridges with β-catenin K435 and K312 respectively, while F71 packed against the aliphatic chain of R386. To determine whether these residues were essential for the interaction with β-catenin, we generated various ICAT mutants harboring single, double or triple amino-acid substitutions (Table 1). D66 was changed into Glycine (D66G), F71 into Alanine (F71A) and E75 into valine (E75V). IP of nuclear endogenous β-catenin in Mel501 cells by ectopically expressed ICAT WT or single mutants, showed no differences between WT and mutants (Fig 6A). On the opposite, double and triple mutants had a lower affinity (reduction over 70%) for β-catenin than ICAT WT (Fig 6B). Consistent with these findings, single mutations of conserved ICAT residues did not affect significantly the luciferase activity of the *M-MITF* promoter in the presence of exogenous LEF1, whereas the double and triple mutations significantly reduced, but did not totally abolish, the negative regulatory role of ICAT (Fig 6C).

### β-catenin/ICAT interaction is diminished but not abolished in mutants K312E, R386G and K435E

**Fig 7:**
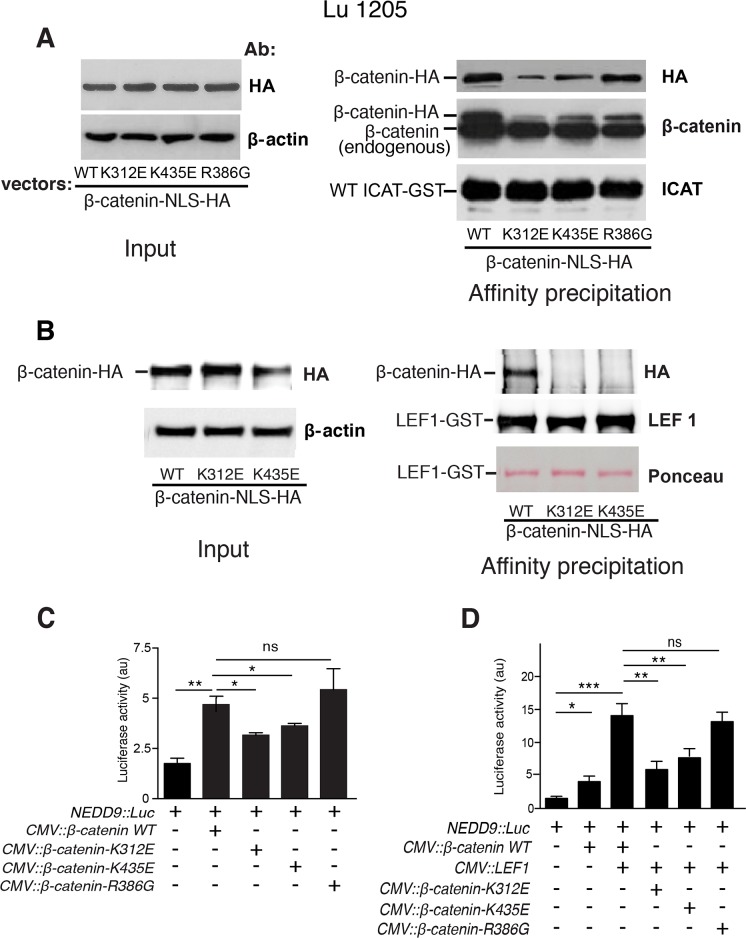
Interactions between ICAT and β-catenin mutants, K312E, K435E and R386G in Lu1205 cell extracts: Consequences on *NEDD9* promoter activity. **A**. Left: WB analysis of lysates (Input) from Lu1205 cells transfected with WT or mutant HA-tagged β-catenin constructs; right: Pull-down assay of HA-tagged WT and mutant β-catenin (K312E, K435E and R386G) by WT ICAT-GST recombinant protein. **B**. Left: WB analysis of lysates (Input) from Lu1205 cells transfected with WT or mutant HA-tagged β-catenin constructs; right: Pull-down assay of HA-tagged WT and mutant β-catenin (K312E and K435E) by LEF1-GST recombinant protein. **C**. Lu1205 cells were transiently transfected with *NEDD9*::*luciferase* and either *β-catenin-WT* or *β-catenin* mutants *(K312E*, *K435E and R386G)* expression vectors. **D**. Lu1205 cells were transiently transfected with *NEDD9*::*luciferase* vector in the presence of *CMV*::*LEF1*. Cells were also transfected with *β-catenin-WT* or *β-catenin* mutants *(K312E*, *K435E and R386G)* expression vectors. Data are presented as means ± SEM of three independent experiments. *p<0.05, **p<0.01, ***p<0.001, ns = not significant.

In order to validate our findings, the facing β-catenin basic residues (K312, K435 and R386) were converted into Glutamate and Glycine, respectively. The effects of these mutations on the interaction with WT ICAT, was assessed through a GST pull-down assay in Lu1205 cells (Fig 7A). K to E mutations markedly decreased, but did not totally suppress the affinity of β-catenin mutants for WT ICAT-GST protein and R386G conversion had no significant effect on this affinity. Likewise, the interaction between β-catenin mutants (K312E and K435E) and LEF1 was tested using recombinant LEF1-GST. No detectable interaction was observed with these mutants (Fig 7B). Luciferase assays were also performed to monitor the interaction between β-catenin mutants and LEF1. Consistent with pull-down results, β-catenin mutants K312E and K435E had a much lower co-transcriptional activity on the *NEDD9* promoter than the WT, both in the absence (Fig 7C) or presence of exogenous LEF1 (Fig 7D), whereas mutant R386G behaved similarly to WT.

We conclude that electrostatic interactions between β-catenin K312 or K435 residues, and the ICAT consensus peptide are important but not crucial for regulating β-catenin co-transcriptional function. Converting one of these attractive electrostatic interactions into repulsion was not fully deleterious and removal of arginine side chain in the R386G mutant was ineffective.

## Discussion

Appropriate Wnt/β-catenin signaling in cells and tissues relies on a subtle balance between positive and negative β-catenin regulators, among which ICAT [15, 35, 36]. In this study, ICAT overexpression in melanoma cells was found to negatively regulate *M-MITF* and *NEDD9* promoter activities, both in the presence and absence of ectopic LEF1. This indicates that in Lu1205 and Mel501 cells, ICAT competes with LEF1 for binding to β-catenin and interferes with its co-transcriptional function.

Simultaneous mutation of the three ICAT residues, Y15, K19 and V22, in the N-terminal domain, predicted to form a hydrophobic patch with β-catenin F660, completely disrupted the ICAT/β-catenin interaction, making this mutant unable to compete with LEF1. Other ICAT residues (M29 and E37) were surmised to establish either hydrophobic interactions (M29) or form a salt bridge (E37) between ICAT and β-catenin [22, 27]. However, simultaneous mutation of residues Y15, K19 and V22, proved to be sufficient to completely suppress the high affinity anchor between the two proteins without impairing the ICAT helical secondary structure. This suggests that M29 and E37 as such, are insufficient to stabilize the complex.

Importantly, β-catenin mutant F660S interacted normally with LEF1 and its co-transcriptional activity on the NEDD9 promoter was equivalent to WT even though it did not bind to ICAT (Fig 4). We conclude that residue F660 is essential for ICAT anchoring but plays no role in the β-catenin interaction with LEF1. Support to this assertion arises from the β-catenin/ICAT crystal structure (S5 Fig) indicating that the interaction relies not only on previously mentioned hydrophobic contacts [22], but also involves a surface complementarity between β-catenin F660 and ICAT Y15, K19 and V22. Residues Y15 and V22 seem to act like pincers around F660, thus rendering this interaction stronger than a mere hydrophobic contact.

Conserved residues (D66, F71, E75) in the ICAT consensus peptide, also present in the catenin-binding domain of TCF7L2 and LEF1 (Fig 1B), have been reported to be crucial for the interaction with β-catenin Arm repeats 5–9. Owing to conflicting reports using mutated TCF7L2 consensus residues [28] or TCF7L2 synthetic peptides [29], the precise contribution of residue F21 (F26 in LEF1 or F71 in ICAT) on β-catenin affinity for TCF/LEF factors, was questioned. Our results indicate that the F71A ICAT mutant behaves similarly to the WT protein (Fig 6B). Close examination of β-catenin/ICAT crystal structures (Fig 1C) reveals that the contact between ICAT F71 aromatic ring and the aliphatic portion of the β-catenin R386 side chain is not sufficient to create a stable hydrophobic interaction, owing to partial exposure of F71 to the solvent. These observations bring support to the conclusion that F21 residue from TCF7L2 is not a major player in the binding to β-catenin [28]. However, double or triple ICAT mutants had less affinity for β-catenin than WT, suggesting a cumulative contribution of residues D66, F71 and E75 in stabilizing the protein complex. Although these residues are directly involved in the competition with LEF1, their mutation does not completely restore the co-transcriptional activity of WT β-catenin (Fig 6C). Overall, this indicates that even though residues Y15, K19, V22 in the N-terminal domain do not compete with LEF1 for ICAT binding to β-catenin, they play a more critical role in the anchoring process than residues D66, F71 and E75.

**Fig 8:**
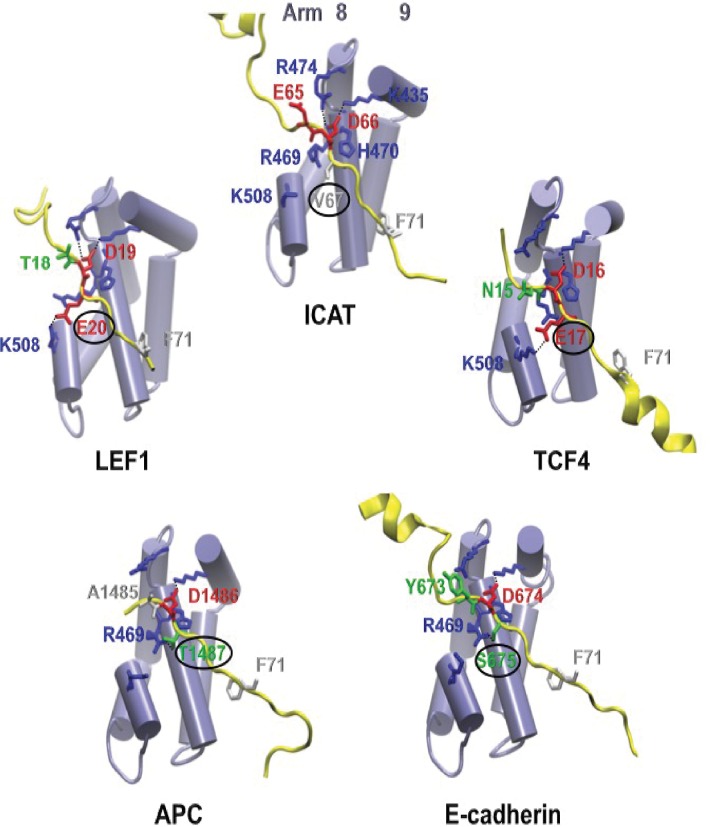
The characteristics of the first X residue in the consensus peptide of several β-catenin binding proteins regulate their interactions with β-catenin. **A**. Zoom is made on the first conserved Aspartate residue of the consensus peptide and its adjacent non conserved residues (shown as sticks) in ICAT, LEF1, TCF4, APC and E-cadherin (yellow ribbons) and the facing β-catenin Arm repeats 8 and 9 (purple cylinders). The first X residue of the consensus is encircled because residue numbering diverges between various β-catenin regulators, although they are facing the same β-catenin residues forming a basic patch. Hydrogen bonds between basic β-catenin residues and their counterpart in β-catenin regulators are presented as black dotted lines. In ICAT, V67 does not establish any hydrogen bond, whereas in LEF1 and TCF4/TCF7L2, E20 and E17, respectively make an H-bond with the facing β-catenin K508. In APC and E-cadherin, T1487 and S675 respectively form hydrogen bonds with the facing β-catenin R469. The color scheme of stick residues based on their characteristics is the same as in Fig 1. PDB codes: ICAT (1luj), LEF1 (3ouw), TCF3/TCF7L1 (1g3j), TCF4/TCF7L2 (1jdh), APC (1t08) and E-cadherin (1i7w).

The affinity of β-catenin for ICAT was shown by isothermal calorimetry to be higher than for LEF1 or APC [27], however when the interaction between the ICAT N-terminal domain and β-catenin is lost, ICAT fails to compete with LEF1 (Fig 3). This suggests that ICAT C-terminal consensus peptide has a weaker affinity for β-catenin than LEF1. Comparison of the consensus peptides from β-catenin partners including TCFs, APC, E-cadherin, LEF1 and ICAT (Fig 1B) reveals the absence in ICAT of hydrophilic residues between D and Φ in the DXθθXΦX_2-7_E sequence, thus accounting for its lower affinity. When the first X residue is hydrophilic, it forms a hydrogen bond with β-catenin R469 or K508 (Fig 8). The second X is generally hydrophobic, except in APC where H1490 interacts with β-catenin D390. Finally, residues X_2-7_ do not generally interact with β-catenin, at least in the complexes whose structures are available in PDB (Fig 8). Comparative analysis of the ICAT consensus sequences from several vertebrates reveals that the first X in this sequence, is predominantly a valine (more scarcely a glycine), and the second X is always an alanine. Therefore, unlike TCF/LEF, APC and E-cadherin, the tightening of the interaction between the ICAT C-terminal domain and β-catenin positive groove by additional hydrogen bonds is not possible, resulting in a weaker interaction. This weakness is probably required for efficient competition with other regulators.

Based on these observations, the dynamic process of ICAT binding to β-catenin may involve one or two steps. The one step process is unlikely because the N-terminal domain is intrinsically structured while the C-terminal domain is unstructured. Consequently, the N-terminal domain should bind more rapidly than the C-terminal domain since during this process the loss of entropy of the N-terminal domain alone (TΔS = -1.5 Kcal/mol) was less unfavorable than that of the full length protein (TΔS = -8.0 kcal/mol) [27]. Therefore, binding of ICAT full length to β-catenin would presumably occur in two steps: i) the ICAT helical N-terminal domain first anchors to β-catenin through F660, then, ii) the ICAT unstructured C-terminal domain lies into the groove and become structured resulting in an increased negative entropy change [27].

## Conclusion

The Wnt/β-catenin signaling is regulated, among others, by ICAT. This process implies a competition between ICAT and various β-catenin ligands sharing a consensus peptide that mediates, with variable affinity, the interaction with β-catenin Arm 5–9. Differences in affinity seem to depend not only on conserved residues in ligands but also on non-conserved ones. ICAT being a negative regulator, the affinity of its consensus peptide needs to be lower than that of positive β-catenin interactors to facilitate its removal. However, ICAT, through its N-terminal domain, holds its capacity to bind β-catenin residue F660 located more than 30 Å apart from its consensus peptide. Loss of both hydrophobicity and surface complementarity of this unique residue (F660) completely prevents ICAT from achieving its negative regulation.

## Supporting information

S1 Fig Crystal structure of human β-catenin Arm repeat region and C-terminal domain. Helix C (in red) in the C-terminal domain runs parallel to helix 3 of Arm repeat 12 (PDB code 2Z6H). Residue F660 in helix 3 (in yellow) is in close contact with residues L674 and L678 (in red) in helix C. Figure was drawn using VMD software [38]
(PDF)

S2 Fig Model for the competition between ICAT and LEF1 for binding to β-catenin based on structural data. The central core of β-catenin is made of 12 armadillo (arm) repeats (in blue) and an additional helical domain, helix C (in red) that docks to the 12^th^ arm repeat [20]. The N-terminal and the C-terminal domains (in white) are unstructured. β-catenin residues predicted to form electrostatic (K312 and K435) or hydrophobic interactions (R386) with LEF1 residues D21, E29 and F26 are shown as blue arrowheads. ICAT and LEF1 residues reported to have putatively the most critical contribution to the interaction with β-catenin are shown as arrowheads (blue for β-catenin, purple for ICAT and orange for LEF1). These interactions are either hydrophobic (represented by green dotted lines) or hydrophilic (represented by black dotted lines). The HMG box of LEF1 interacts with the TCF/LEF binding element (TBE) in the promoter of target genes.
(PDF)

S3 Fig β-catenin, MITF and LEF1 are differently expressed in Mel501 and Lu1205 melanoma cells. **A**. WB (upper panel) and IF (lower panel) analyses of Mel501 and Lu1205 cells: The non phospho S33/37/T41 active form of β-catenin (ABC) is much more abundant (7 fold) in Mel501 than in Lu1205 cells and mainly visible in their nuclei. The numbers below each lane represent normalized densitometry values. α-tubulin = loading control; (bars = 20 μm). **B**. WB (upper panel) and IF (lower panel) analyses of MITF in Mel501 and Lu1205 cells. MITF is present in the nuclei of Mel501 cells. The two bands correspond to at least two different MITF isoforms. Lu1205 cells are devoid of MITF (bars = 10 μm). **C**. qRT-PCR analysis of LEF1 and TCF7L2 mRNA levels in Lu1205 and Mel501 cells. **D.** WB analysis of endogenous LEF1 protein levels in Mel501 and Lu1205 cells. Numbers represent normalized densitometry values. β-actin = loading control.
(PDF)

S4 Fig Circular dichroism (CD) analysis of purified ICAT-WT and DQE recombinant proteins. **A**. Gel electrophoresis and Coomassie blue staining of purified proteins. **B**. Far-UV CD spectra of ICAT WT (in red) and ICAT DQE (in blue) recombinant proteins diluted at 30 μM in 10mM sodium phosphate, 100mM ammonium sulphate buffer pH 7.0. Data were recorded at 20°C. Similar results were obtained with 50 μM protein concentrations. **C.** Thermal denaturation curves of ICAT WT and ICAT DQE. Tm = melting temperature.
(PDF)

S5 Fig Embedding of μ-catenin F660 in the ICAT N-terminal domain. The entire ICAT protein is shown (surface), with its globular N-terminal domain and extended C-terminal domain. The residues are colored according to their characteristics: white for hydrophobic, green for polar, red for acidic and blue for basic residues. β-catenin residue F660, part of Arm repeat 12 helix 3 (purple cylinder) is shown as pink hard spheres. It is embedded in an ICAT niche made of residues Y15, K19 and V22.
(PDF)

S1 Table Primers used to create the different mutants. (DOCX)

S2 Table Results of the yeast two-hybrid screening using CTNNBIP1/ICAT as bait and cDNA from human melanocytes as prey library. * PBS (Prey-Bait-Score) was automatically computed. A and B represent respectively very high and high confidence in the interaction. D represents moderate confidence. N/A = non applicable.
(DOCX)

S3 Table List of CTNNBIP1/ICAT interactors in HEK cells identified by affinity capture coupled to mass spectrometry (MS). Data were compiled from [31]. Human epithelial kidney (HEK) cells were used for affinity capture experiments. *Interactors identified in both studies (cf S2 Table). **Computed confidence score based on partial least squares model with values between 0 and 1. Values higher than 0.3 are considered as high confidence interactions.
(DOCX)

S4 Table Comparative levels of ICAT, β-catenin, MITF and LEF1 proteins in melanoma cells. (DOCX)

## Abbreviations

ICAT: Inhibitor of β-catenin and TCF
TCF: T Cell Factor
Arm: armadillo
MITF: Microphtalmia Transcription Factor
NEDD9: Neural precursor cell Expressed Developmentally Down-regulated 9
WB: Western blot
CD: Circular dichroism

## References

[1] Gallagher SJ, Rambow F, Kumasaka M, Champeval D, Bellacosa A, Delmas V, et al Beta-catenin inhibits melanocyte migration but induces melanoma metastasis. Oncogene. 2013;32: 2230–2238. 10.1038/onc.2012.229 22665063 PMC3637425

[2] Clevers H, Nusse R. Wnt/β-catenin signaling and disease. Cell. 2012;149: 1192–1205. 10.1016/j.cell.2012.05.012 22682243

[3] Anastas JN, Moon RT. WNT signalling pathways as therapeutic targets in cancer. Nat Rev Cancer. 2013;13: 11–26. 10.1038/nrc3419 23258168

[4] Huels DJ, Ridgway RA, Radulescu S, Leushacke M, Campbell AD, Biswas S, et al E-cadherin can limit the transforming properties of activating β-catenin mutations. EMBO J. 2015;34: 2321–2333. 10.15252/embj.201591739 26240067 PMC4570519

[5] Steingrímsson E, Copeland NG, Jenkins NA. Melanocytes and the microphthalmia transcription factor network. Annu Rev Genet. 2004;38: 365–411. 10.1146/annurev.genet.38.072902.092717 15568981

[6] Levy C, Khaled M, Fisher DE. MITF: master regulator of melanocyte development and melanoma oncogene. Trends Mol Med. 2006;12: 406–414. 10.1016/j.molmed.2006.07.008 16899407

[7] Selzer E, Wacheck V, Lucas T, Heere-Ress E, Wu M, Weilbaecher KN, et al The melanocyte-specific isoform of the microphthalmia transcription factor affects the phenotype of human melanoma. Cancer Res. 2002;62: 2098–2103. 11929831

[8] Widlund HR, Horstmann MA, Price ER, Cui J, Lessnick SL, Wu M, et al Beta-catenin-induced melanoma growth requires the downstream target Microphthalmia-associated transcription factor. J Cell Biol. 2002;158: 1079–1087. 10.1083/jcb.200202049 12235125 PMC2173224

[9] Hoek KS, Eichhoff OM, Schlegel NC, Döbbeling U, Kobert N, Schaerer L, et al In vivo switching of human melanoma cells between proliferative and invasive states. Cancer Res. 2008;68: 650–656. 10.1158/0008-5472.CAN-07-2491 18245463

[10] Kim M, Gans JD, Nogueira C, Wang A, Paik J-H, Feng B, et al Comparative oncogenomics identifies NEDD9 as a melanoma metastasis gene. Cell. 2006;125: 1269–1281. 10.1016/j.cell.2006.06.008 16814714

[11] Li Y, Bavarva JH, Wang Z, Guo J, Qian C, Thibodeau SN, et al HEF1, a novel target of Wnt signaling, promotes colonic cell migration and cancer progression. Oncogene. 2011;30: 2633–2643. 10.1038/onc.2010.632 21317929 PMC3164309

[12] Domingues MJ, Rambow F, Job B, Papon L, Liu W, Larue L, et al β-catenin inhibitor ICAT modulates the invasive motility of melanoma cells. Cancer Res. 2014;74: 1983–1995. 10.1158/0008-5472.CAN-13-0920 24514042

[13] Daniels DL, Weis WI. Beta-catenin directly displaces Groucho/TLE repressors from Tcf/Lef in Wnt-mediated transcription activation. Nat Struct Mol Biol. 2005;12: 364–371. 10.1038/nsmb912 15768032

[14] Bottomly D, Kyler SL, McWeeney SK, Yochum GS. Identification of {beta}-catenin binding regions in colon cancer cells using ChIP-Seq. Nucleic Acids Res. 2010;38: 5735–5745. 10.1093/nar/gkq363 20460455 PMC2943592

[15] Mosimann C, Hausmann G, Basler K. Beta-catenin hits chromatin: regulation of Wnt target gene activation. Nat Rev Mol Cell Biol. 2009;10: 276–286. 10.1038/nrm2654 19305417

[16] Tago K, Nakamura T, Nishita M, Hyodo J, Nagai S, Murata Y, et al Inhibition of Wnt signaling by ICAT, a novel beta-catenin-interacting protein. Genes Dev. 2000;14: 1741–1749. 10898789 PMC316784

[17] Satoh K, Kasai M, Ishidao T, Tago K, Ohwada S, Hasegawa Y, et al Anteriorization of neural fate by inhibitor of beta-catenin and T cell factor (ICAT), a negative regulator of Wnt signaling. Proc Natl Acad Sci USA. 2004;101: 8017–8021. 10.1073/pnas.0401733101 15148409 PMC419549

[18] Gottardi CJ, Gumbiner BM. Role for ICAT in beta-catenin-dependent nuclear signaling and cadherin functions. Am J Physiol, Cell Physiol. 2004;286: C747–756. 10.1152/ajpcell.00433.2003 14613891

[19] Huber AH, Nelson WJ, Weis WI. Three-dimensional structure of the armadillo repeat region of beta-catenin. Cell. 1997;90: 871–882. 9298899 10.1016/s0092-8674(00)80352-9

[20] Xing Y, Takemaru K-I, Liu J, Berndt JD, Zheng JJ, Moon RT, et al Crystal structure of a full-length beta-catenin. Structure. 2008;16: 478–487. 10.1016/j.str.2007.12.021 18334222 PMC4267759

[21] Mo R, Chew T-L, Maher MT, Bellipanni G, Weinberg ES, Gottardi CJ. The terminal region of beta-catenin promotes stability by shielding the Armadillo repeats from the axin-scaffold destruction complex. J Biol Chem. 2009;284: 28222–28231. 10.1074/jbc.M109.045039 19706613 PMC2788874

[22] Graham TA, Clements WK, Kimelman D, Xu W. The crystal structure of the beta-catenin/ICAT complex reveals the inhibitory mechanism of ICAT. Mol Cell. 2002;10: 563–571. 12408824 10.1016/s1097-2765(02)00637-8

[23] Daniels DL, Weis WI. ICAT inhibits beta-catenin binding to Tcf/Lef-family transcription factors and the general coactivator p300 using independent structural modules. Mol Cell. 2002;10: 573–584. 12408825 10.1016/s1097-2765(02)00631-7

[24] Graham TA, Weaver C, Mao F, Kimelman D, Xu W. Crystal structure of a beta-catenin/Tcf complex. Cell. 2000;103: 885–896. 11136974 10.1016/s0092-8674(00)00192-6

[25] Poy F, Lepourcelet M, Shivdasani RA, Eck MJ. Structure of a human Tcf4-beta-catenin complex. Nat Struct Biol. 2001;8: 1053–1057. 10.1038/nsb720 11713476

[26] Ha N-C, Tonozuka T, Stamos JL, Choi H-J, Weis WI. Mechanism of phosphorylation-dependent binding of APC to beta-catenin and its role in beta-catenin degradation. Mol Cell. 2004;15: 511–521. 10.1016/j.molcel.2004.08.010 15327768

[27] Choi H-J, Huber AH, Weis WI. Thermodynamics of beta-catenin-ligand interactions: the roles of the N- and C-terminal tails in modulating binding affinity. J Biol Chem. 2006;281: 1027–1038. 10.1074/jbc.M511338200 16293619

[28] Fasolini M, Wu X, Flocco M, Trosset J-Y, Oppermann U, Knapp S. Hot spots in Tcf4 for the interaction with beta-catenin. J Biol Chem. 2003;278: 21092–21098. 10.1074/jbc.M301781200 12657632

[29] Gail R, Frank R, Wittinghofer A. Systematic peptide array-based delineation of the differential beta-catenin interaction with Tcf4, E-cadherin, and adenomatous polyposis coli. J Biol Chem. 2005;280: 7107–7117. 10.1074/jbc.M410215200 15591320

[30] Sreerama N, Woody RW. Estimation of protein secondary structure from circular dichroism spectra: comparison of CONTIN, SELCON, and CDSSTR methods with an expanded reference set. Anal Biochem. 2000;287: 252–260. 10.1006/abio.2000.4880 11112271

[31] Ewing RM, Chu P, Elisma F, Li H, Taylor P, Climie S, et al Large-scale mapping of human protein-protein interactions by mass spectrometry. Mol Syst Biol. 2007;3: 89 10.1038/msb4100134 17353931 PMC1847948

[32] Williams BO, Barish GD, Klymkowsky MW, Varmus HE. A comparative evaluation of beta-catenin and plakoglobin signaling activity. Oncogene. 2000;19: 5720–5728. 10.1038/sj.onc.1203921 11126358

[33] Carreira S, Goodall J, Denat L, Rodriguez M, Nuciforo P, Hoek KS, et al Mitf regulation of Dia1 controls melanoma proliferation and invasiveness. Genes Dev. 2006;20: 3426–3439. 10.1101/gad.406406 17182868 PMC1698449

[34] Mao CD, Byers SW. Cell-context dependent TCF/LEF expression and function: alternative tales of repression, de-repression and activation potentials. Crit Rev Eukaryot Gene Expr. 2011;21: 207–236. 22111711 10.1615/critreveukargeneexpr.v21.i3.10 PMC3434703

[35] Yang P-T, Anastas JN, Toroni RA, Shinohara MM, Goodson JM, Bosserhoff AK, et al WLS inhibits melanoma cell proliferation through the β-catenin signalling pathway and induces spontaneous metastasis. EMBO Mol Med. 2012;4: 1294–1307. 10.1002/emmm.201201486 23129487 PMC3531604

[36] Jung H-Y, Jun S, Lee M, Kim H-C, Wang X, Ji H, et al PAF and EZH2 induce Wnt/β-catenin signaling hyperactivation. Mol Cell. 2013;52: 193–205. 10.1016/j.molcel.2013.08.028 24055345 PMC4040269

[37] Frishman D, Argos P. Knowledge-based protein secondary structure assignment. Proteins. 1995;23: 566–579. 10.1002/prot.340230412 8749853

[38] Humphrey W, Dalke A, Schulten K. VMD: visual molecular dynamics. J Mol Graph. 1996;14: 33–38, 27–28. 8744570 10.1016/0263-7855(96)00018-5

