# Supplementary figures and images for: Structure-based mutational analysis of ICAT residues mediating negative regulation of β-catenin co-transcriptional activity
Source: PLoS One. 2017 Mar 8;12(3):e0172603. doi: 10.1371/journal.pone.0172603 (PMC5342195; doi:10.1371/journal.pone.0172603)

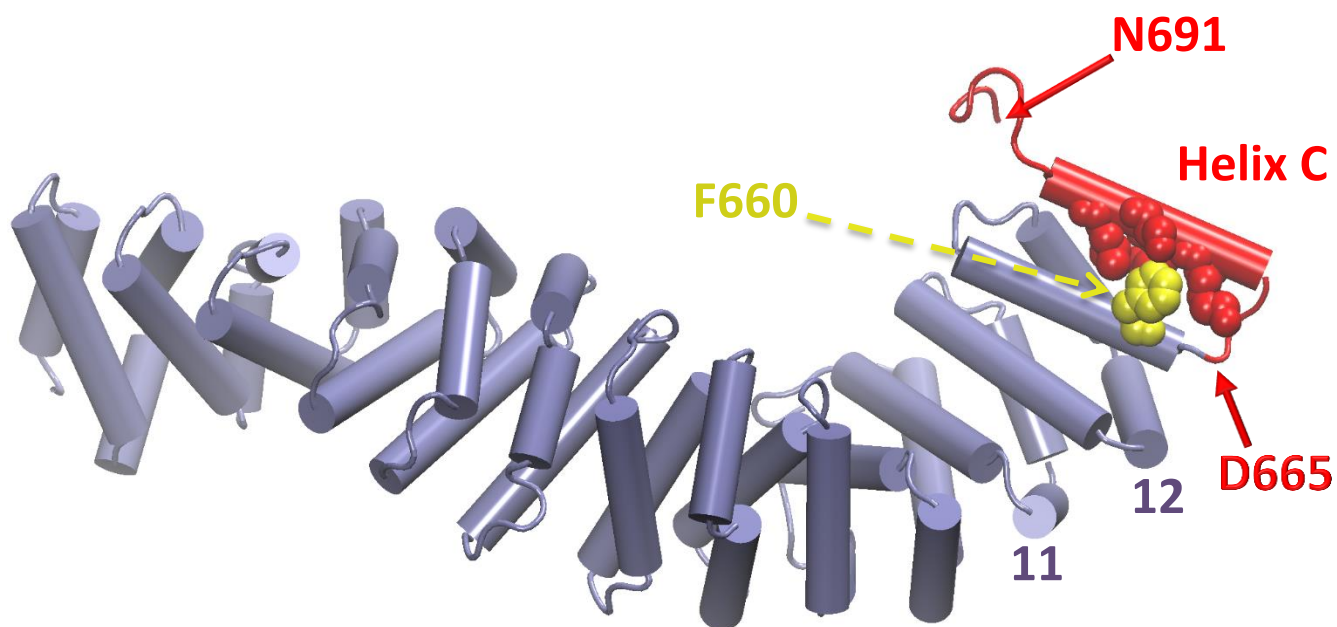

Domingues\_Fig S1

Supplement: S1 Fig — Helix C (in red) in the C-terminal domain runs parallel to helix 3 of Arm repeat 12 (PDB code 2Z6H). Residue F660 in helix 3 (in yellow) is in close contact with residues L674 and L678 (in red) in helix C. Figure was drawn using VMD software [38] (PDF) [file pone.0172603.s001.pdf]

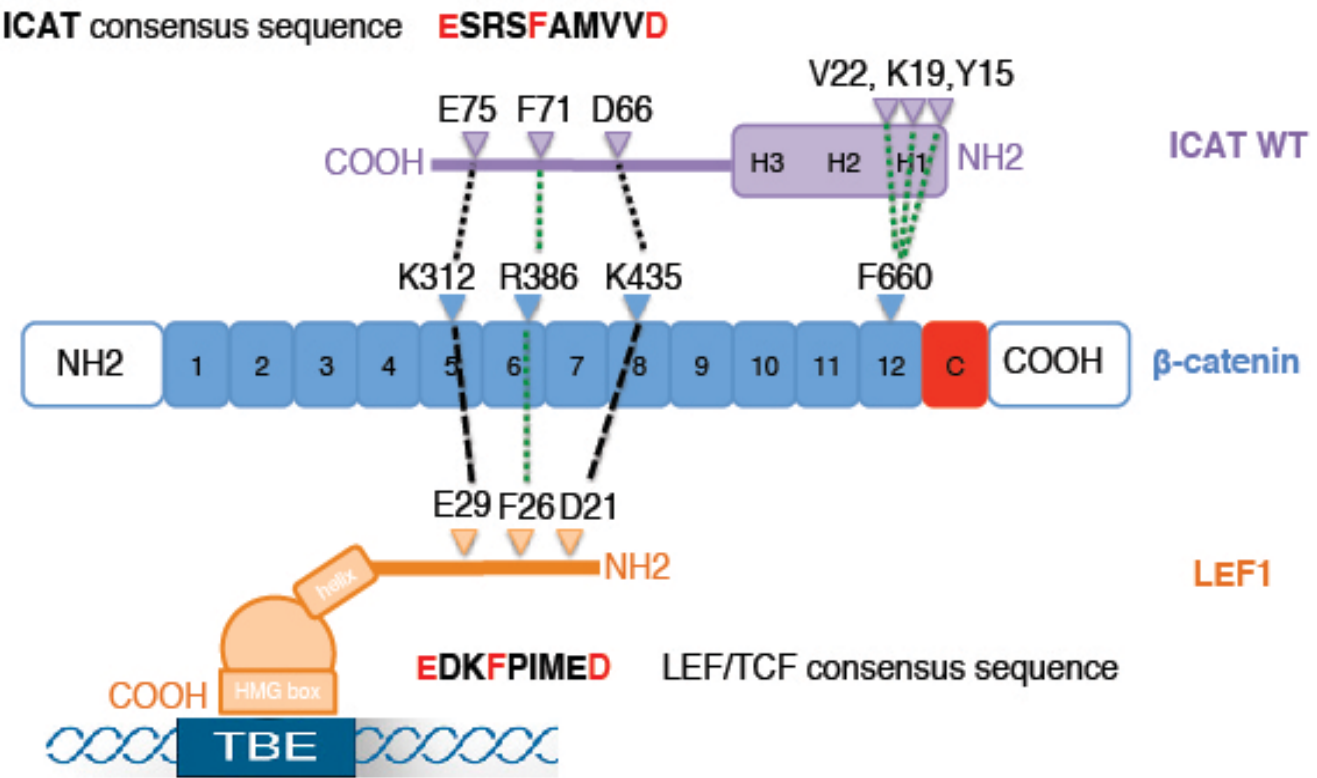

Domingues\_Fig S2

Supplement: S2 Fig — The central core of β-catenin is made of 12 armadillo (arm) repeats (in blue) and an additional helical domain, helix C (in red) that docks to the 12th arm repeat [20]. The N-terminal and the C-terminal domains (in white) are unstructured. β-catenin residues predicted to form electrostatic (K312 and K435) or hydrophobic interactions (R386) with LEF1 residues D21, E29 and F26 are shown as blue arrowheads. ICAT and LEF1 residues reported to have putatively the most critical contribution to the interaction with β-catenin are shown as arrowheads (blue for β-catenin, purple for ICAT and orange for LEF1). These interactions are either hydrophobic (represented by green dotted lines) or hydrophilic (represented by black dotted lines). The HMG box of LEF1 interacts with the TCF/LEF binding element (TBE) in the promoter of target genes. (PDF) [file pone.0172603.s002.pdf]

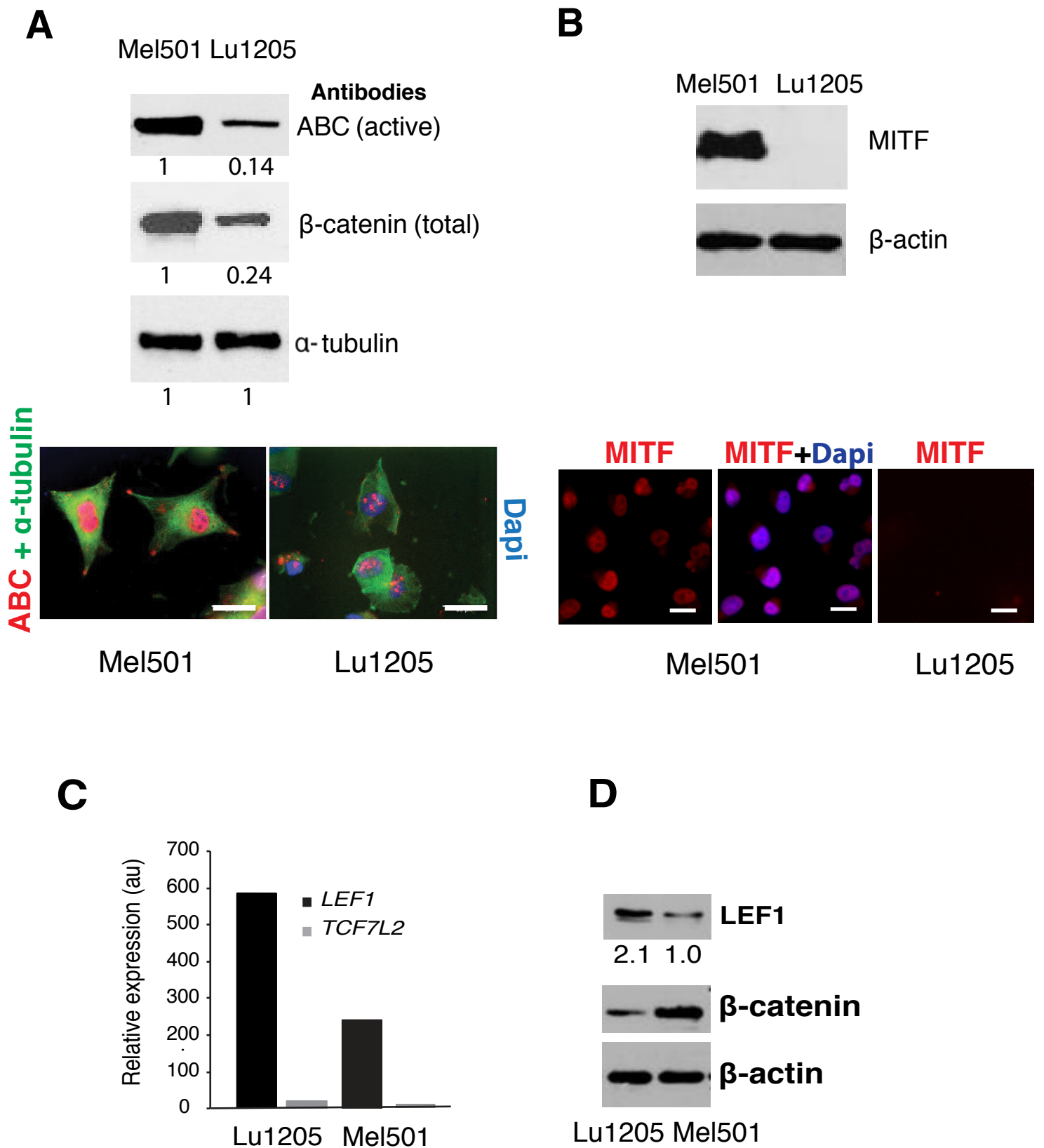

Supplement: S3 Fig — A. WB (upper panel) and IF (lower panel) analyses of Mel501 and Lu1205 cells: The non phospho S33/37/T41 active form of β-catenin (ABC) is much more abundant (7 fold) in Mel501 than in Lu1205 cells and mainly visible in their nuclei. The numbers below each lane represent normalized densitometry values. α-tubulin = loading control; (bars = 20 μm). B. WB (upper panel) and IF (lower panel) analyses of MITF in Mel501 and Lu1205 cells. MITF is present in the nuclei of Mel501 cells. The two bands correspond to at least two different MITF isoforms. Lu1205 cells are devoid of MITF (bars = 10 μm). C. qRT-PCR analysis of LEF1 and TCF7L2 mRNA levels in Lu1205 and Mel501 cells. D. WB analysis of endogenous LEF1 protein levels in Mel501 and Lu1205 cells. Numbers represent normalized densitometry values. β-actin = loading control. (PDF) [file pone.0172603.s003.pdf]

**A**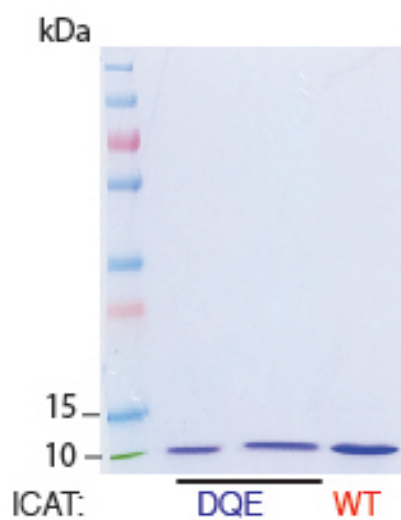**B**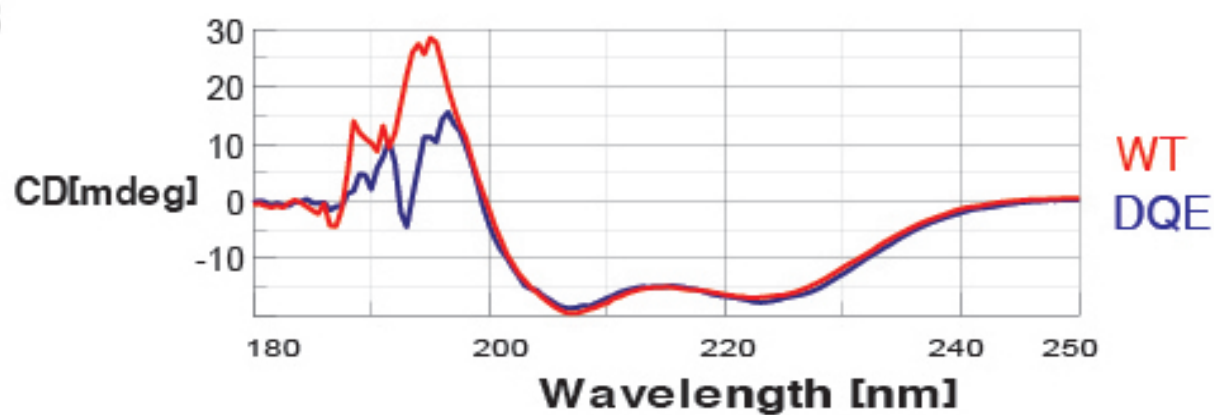**C**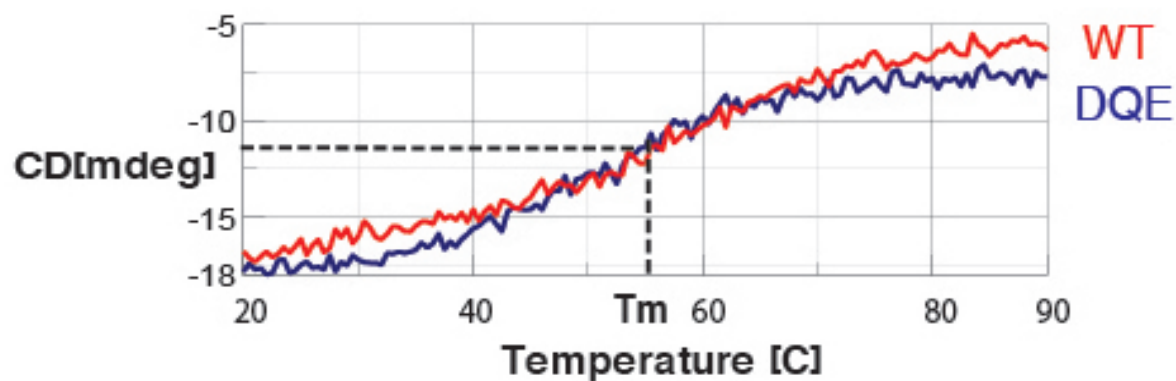

Domingues\_Fig S4

Supplement: S4 Fig — A. Gel electrophoresis and Coomassie blue staining of purified proteins. B. Far-UV CD spectra of ICAT WT (in red) and ICAT DQE (in blue) recombinant proteins diluted at 30 μM in 10mM sodium phosphate, 100mM ammonium sulphate buffer pH 7.0. Data were recorded at 20°C. Similar results were obtained with 50 μM protein concentrations. C. Thermal denaturation curves of ICAT WT and ICAT DQE. Tm = melting temperature. (PDF) [file pone.0172603.s004.pdf]

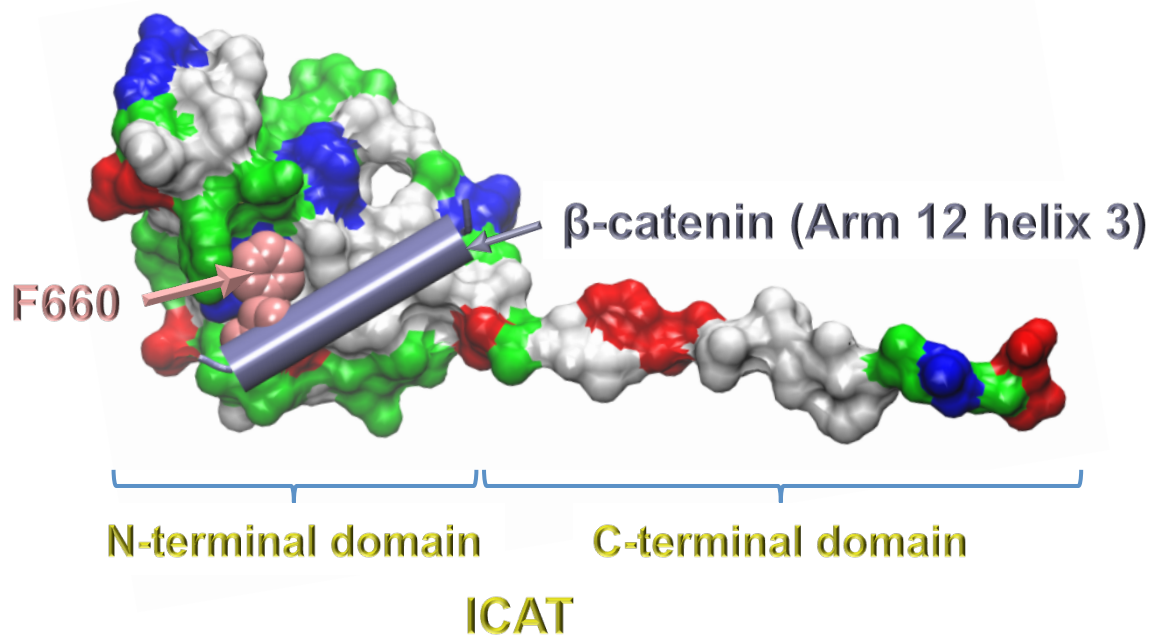

Domingues\_Fig S5

Supplement: S5 Fig — The entire ICAT protein is shown (surface), with its globular N-terminal domain and extended C-terminal domain. The residues are colored according to their characteristics: white for hydrophobic, green for polar, red for acidic and blue for basic residues. β-catenin residue F660, part of Arm repeat 12 helix 3 (purple cylinder) is shown as pink hard spheres. It is embedded in an ICAT niche made of residues Y15, K19 and V22. (PDF) [file pone.0172603.s005.pdf]
