# Supplementary material for: Structure-based mutational analysis of ICAT residues mediating negative regulation of β-catenin co-transcriptional activity
Source: PLoS One. 2017 Mar 8;12(3):e0172603. doi: 10.1371/journal.pone.0172603 (PMC5342195; doi:10.1371/journal.pone.0172603)
Supplement: S1 Table — (DOCX) [file pone.0172603.s006.docx]

**S1 Table.**

| **Gene** | **Primer name** | **Mutation name** | Sequence 5’-3’ |
| --- | --- | --- | --- |
| ICAT  (CTNNBIP1) | LL1806 | DQE | AAG AGT CCG GAG GAG ATG GAC ATT CAG CAG CAG GTC CGA GAG |
|  | LL1521 |  | cgc cat cac cac gcc ctc tgc acc ctg g |
| ICAT | LL1520 | D66G | c cag ggt gca gag ggc gtg gtg atg gcg |
|  | LL1521 |  | cgc cat cac cac gcc ctc tgc acc ctg g |
| ICAT | LL1924 | F71A | gac gtg gtg atg gcg gct tcc agg tcg gag acg |
|  | LL1925 |  | cgt ctc cga cct gga agc cgc cat cac cac gtc |
| ICAT | LL1522 | E75V | g ttt tcc agg tcg gtg acg gaa gac cgg ag |
|  | LL1523 |  | ctc cgg tct tcc gtc acc gac ctg gaa aac |
| ICAT | LL1520 | GV | cca ggg tgca gag ggc gtg gtg atg gcg |
|  | LL1521 |  | cgc cat cac cac gcc ctc tgc acc ctg g |
| ICAT | LL1520 | GA | cca ggg tgca gag ggc gtg gtg atg gcg |
|  | LL1521 |  | cgc cat cac cac gcc ctc tgc acc ctg g |
| ICAT | LL2214 | AV | G GTG ATG GCG GCT TCC AGG TCG GTG ACG GAA GAC CGG |
|  | LL2215 |  | CCG GTC TTC CGT CAC CGA CCT GGA AGC CGC CAT CAC C |
| ICAT | LL1522 | GAV  (with plasmid ICAT-GA) | g ttt tcc agg tcg gtg acg gaa gac cgg ag |
|  | LL1523 |  | ctc cgg tct tcc gtc acc gac ctg gaa aac |
| β-catenin  (CTNNB1) | LL1913 | K312E | GC AAC CAA GAA AGC GAG CTC ATC ATA CTG G |
|  | LL1914 |  | CCA GTA TGA TGA GCT CGC TTT CTT GGT TGC |
| β-catenin | LL1915 | K435E | GC AAT AAT TAT AAG AAC GAG ATG ATG GTC TGC C |
|  | LL1916 |  | ggc aga cca tca tct cgt tct tat aat tat tgc |
| β-catenin | LL2262 | R386G | GT CTT TGG ACT CTC GGG AAT CTT TCA GAT GCT GC |
|  | LL2263 |  | GCA GCA TCT GAA AGA TTC CCG AGA GTC CAA AGAC |
| β-catenin | LL1899 | F660S | GCA GCT GCT GTT TTG TCC CGA ATG TCT GAG GAC |
|  | LL1900 |  | GTC CTC AGA CAT TCG GGA CAA AAC AGC AGC TGC |
| β-catenin | LL2104 | F660A | GCA GCT GCT GTT TTG GCC CGA ATG TCT GAG GAC |
|  | LL2105 |  | GTC CTC AGA CAT TCG GGC CAA AAC AGC AGC TGC |
