# Supplementary material for: Structure-based mutational analysis of ICAT residues mediating negative regulation of β-catenin co-transcriptional activity
Source: PLoS One. 2017 Mar 8;12(3):e0172603. doi: 10.1371/journal.pone.0172603 (PMC5342195; doi:10.1371/journal.pone.0172603)
Supplement: S2 Table — * PBS (Prey-Bait-Score) was automatically computed. A and B represent respectively very high and high confidence in the interaction. D represents moderate confidence. N/A = non applicable. (DOCX) [file pone.0172603.s007.docx]

S2 Table

| **Gene** (human) | **Accession number**  (NCBI) | **Interactor protein**  (prey) | **Prey Clones**  (% of total) PBS* |
| --- | --- | --- | --- |
| AFF4 | NM_014423 | AFF4 (RNA binding  Protein) | 4 (1.6%)  **B** |
| AP1G1 | NM_001030007 | Gamma - Adaptin | 6 (2.4%)  **B** |
| CTNNB1 | NM_0202248.2 | β-catenin | 198 (79.2%)  **A** |
| IFIT3 | NM_001549 | Interferon-induced  protein RIG-G | 4 (1.6%)  **B** |
| IFIT5 | NM_012420 | Interferon- induced  protein RI58 | 10 (4%)  **A** |
| JUP | NM_002230 | Junction plakoglobin  (γ-catenin) | 1 (0.4%)  **N/A** |
| PPP2R5D | NM_006245 | Protein phosphatase2 (subunit5) | 4 (1.6%)  **B** |
| STK24 | NM_003576 | Serine/Threonine  kinase Mst3 | 11 (4.4%)  **A** |
| TIMM8A | NM_011734 | Translocase | 10 (4%)  **A** |
| UPS47 | NM_001282659.1 | Ubiquitin specific peptidase 47 | 1 (0.4%)  **D** |
| USP9X | NM_001039591.2 | Ubiquitin specific peptidase 9 X-linked | 1 (0.4%)  **D** |
