# Supplementary material for: Structure-based mutational analysis of ICAT residues mediating negative regulation of β-catenin co-transcriptional activity
Source: PLoS One. 2017 Mar 8;12(3):e0172603. doi: 10.1371/journal.pone.0172603 (PMC5342195; doi:10.1371/journal.pone.0172603)
Supplement: S3 Table — Data were compiled from [31]. Human epithelial kidney (HEK) cells were used for affinity capture experiments. *Interactors identified in both studies (cf S2 Table). **Computed confidence score based on partial least squares model with values between 0 and 1. Values higher than 0.3 are considered as high confidence interactions. (DOCX) [file pone.0172603.s008.docx]

**S3 Table.**

| **Gene**  (human) | **Accession number**  (NCBI) | **Interactor protein** | **Score**** |
| --- | --- | --- | --- |
| CASP4 | NM_033306.2 | Caspase 4 | 0.316 |
| CPVL | NM_031311.3 | Carboxypeptidase  Vitellogenic-like | 0.4 |
| CTNNB1* | NM_02022482 | β-catenin | 0.545 |
| CTSA | NM_000308.3 | Cathepsin A | 0.384 |
| GLB1 | NM_000404.3 | β-1 Galactosidase | 0.544 |
| GOT2 | NM_002080.3 | Nascent polypeptide  Associated complex | 0.392 |
| JUP* | NM_002230 | Junction plakoglobin  (γ-catenin) | 0.454 |
| NACAP1 | NR_002182.1 | Non coding RNA | 0.392 |
| PNMA2 | NM_007257.5 | Paraneoplastic Ma  antigen 2 | 0.394 |
| PPP2R1A | NM_014225.5 | Protein phosphatase 2 regulatory subunit A | 0.318 |
| RPA3 | NM_002947.3 | Replication protein A3,14 kDa | 0.343 |
