# Supplementary material for: Structure-based mutational analysis of ICAT residues mediating negative regulation of β-catenin co-transcriptional activity
Source: PLoS One. 2017 Mar 8;12(3):e0172603. doi: 10.1371/journal.pone.0172603 (PMC5342195; doi:10.1371/journal.pone.0172603)
Supplement: S4 Table — (DOCX) [file pone.0172603.s009.docx]

| **Cell line** | **ICAT levels** | **β-catenin levels** | **MITF levels** | **LEF1 levels** |
| --- | --- | --- | --- | --- |
| Mel501 | + | ++++ | +++ | + |
| Lu1205 | +++ | + | - | ++ |

**S4 Table**
